# ZNF114 as a prognostic biomarker associated with Wnt/EMT and immune-related features in gastric cancer

**DOI:** 10.3389/fonc.2026.1946670

**Published:** 2026-10-20

**Authors:** Wenzheng Li, Zhengxuan Zhang, Yiyan Li, Aoxue Xing, Hailiang Wang, Gang Wu, Peichun Sun, Wei Zhang

**Affiliations:** 1 Henan Provincial People’s Hospital, Henan Provincial Research Center for Precision Control Engineering of Digestive Tract Tumors, Zhengzhou, China; 2 Department of Gastrointestinal Surgery, People’s Hospital of Henan University, Henan Provincial People’s Hospital, School of Clinical Medicine, Henan University, Zhengzhou, Henan, China; 3 Department of Gastrointestinal Surgery, People’s Hospital of Zhengzhou University, Henan Provincial People’s Hospital, School of Clinical Medicine, Zhengzhou University, Zhengzhou, Henan, China

**Keywords:** epithelial-mesenchymal transition, gastric cancer, immune-related transcriptomic features, prognosis, Wnt signaling, ZNF114

## Abstract

**Objective:**

To investigate ZNF114 expression, clinical significance, and its role in malignant phenotypes and its associations with immune-related features in gastric cancer (GC).

**Methods:**

ZNF114 expression and prognosis were analyzed using The Cancer Genome Atlas (TCGA) stomach adenocarcinoma (STAD) and Gene Expression Omnibus (GEO) datasets (GSE79973, GSE65801, GSE13861), and validated in an in-house cohort of six paired gastric cancer and adjacent non-tumor specimens via Western blotting and immunohistochemistry. ZNF114-knockdown GC cells were established using small interfering RNA (siRNA). Rescue experiments used an si-2-resistant ZNF114 construct in AGS and HGC-27 cells. Cell Counting Kit-8 (CCK-8), colony formation, flow cytometry, wound healing, Transwell assays, gene set enrichment analysis (GSEA) and Western blotting were used to evaluate proliferation, apoptosis, migration, invasion and Wnt/epithelial-mesenchymal transition (EMT)-associated signaling. A xenograft model was used for *in vivo* validation, with Ki-67 and terminal deoxynucleotidyl transferase dUTP nick end labeling (TUNEL) staining. Immune microenvironment analysis included immune-cell/signature scoring, checkpoint-landscape evaluation, tumor mutational burden (TMB) and genome-feature assessment, and exploratory drug-sensitivity prediction using oncoPredict based on GDSC2 data.

**Results:**

ZNF114 was overexpressed in GC tissues and cell lines, with high expression linked to poor survival and adverse clinicopathology. ZNF114 knockdown inhibited proliferation, migration and invasion, and induced apoptosis. ZNF114 was associated with EMT and Wnt signaling; its silencing increased E-cadherin and decreased N-cadherin, Vimentin and the Wnt-related proteins β-catenin, c-Myc, Cyclin D1 and p-GSK3β. Re-expression of ZNF114 attenuated the knockdown-associated EMT changes and restored malignant phenotypes in rescue assays. High ZNF114 expression was associated with elevated Wnt/EMT signaling and a higher composite immune-cold index (FDR < 0.001), alongside altered non-classical checkpoint patterns, lower tumor mutational burden and predicted drug-sensitivity differences, whereas individual immune-activation features showed non-significant downward trends after multiple-testing correction. These computational findings are TCGA-based, associative and hypothesis-generating. *In vivo*, ZNF114 knockdown suppressed tumor growth.

**Conclusion:**

ZNF114 is associated with malignant phenotypes and Wnt/EMT-associated remodeling. Its high expression is associated with a higher composite immune-cold index and altered immune-related transcriptomic features, supporting ZNF114 as a candidate prognostic biomarker and a candidate for future mechanistic and therapy-oriented studies.

## 1 Introduction

Gastric cancer (GC) is one of the most prevalent and lethal malignancies of the digestive system worldwide (1). Despite substantial advances in multimodal treatment strategies centered on surgery and combined with targeted therapy and immunotherapy, the overall prognosis of patients with advanced gastric cancer remains poor, as most cases are diagnosed at an advanced stage and are frequently accompanied by local invasion, lymph node metastasis, or distant metastasis (2). Therefore, a deeper understanding of the molecular mechanisms driving gastric cancer invasion, metastasis, and immune evasion, as well as the identification of novel biomarkers and therapeutic targets, is of considerable scientific and clinical importance.

The zinc finger protein (ZFP) family constitutes one of the largest groups of transcription factors in the human genome and participates extensively in a wide range of biological processes, including cell proliferation, differentiation, apoptosis, and the maintenance of genomic stability, through specific interactions with DNA, RNA, or proteins (3). Accumulating evidence has demonstrated that ZFPs play critical regulatory roles in the development and progression of gastric cancer; however, the functions of individual family members appear to be highly heterogeneous. Some ZFPs promote tumor cell proliferation, migration, invasion, and therapeutic resistance by activating key signaling pathways (4, 5), whereas others exhibit tumor-suppressive effects due to promoter hypermethylation-mediated silencing (6, 7). These observations suggest that further investigation of distinct ZFP family members may provide important insights into the molecular basis of gastric cancer pathogenesis. ZNF114 is a member of the C2H2-type zinc finger protein family. Previous studies have indicated that ZNF114 may have clinical relevance in several solid tumors (15, 16); however, its expression profile, prognostic significance, and biological functions in gastric cancer remain largely unclear.

Epithelial–mesenchymal transition (EMT) is a critical process by which tumor cells acquire migratory and invasive capacities and represents one of the key molecular bases underlying gastric cancer metastasis (8, 25). Earlier work has shown that certain transcriptional regulators can drive the EMT program and promote malignant progression by modulating the expression of epithelial markers such as E-cadherin and mesenchymal markers such as N-cadherin and Vimentin (9, 10). Meanwhile, EMT is not only closely associated with tumor invasion and metastasis but is also intricately linked to remodeling of the tumor immune microenvironment. In gastric cancer, active EMT is often accompanied by reduced infiltration of cytotoxic T cells, increased expression of immune checkpoint-related molecules, and enhanced recruitment of immunosuppressive cells, thereby contributing to the formation of a tumor microenvironment with more pronounced immune-evasive features (11–13). Notably, members of the zinc finger protein family have also been implicated in this regulatory process. For example, ZNF207 has been reported to be associated with immune microenvironment remodeling and immune evasion in hepatocellular carcinoma (14). These observations motivated us to investigate whether ZNF114 is associated with EMT and immune-related features in gastric cancer. Furthermore, Wnt/β-catenin signaling has been increasingly recognized as a critical upstream driver of EMT in gastric cancer, and several C2H2-type zinc finger proteins have been shown to modulate Wnt pathway activity (24). Recent studies have also begun to distinguish classical T-cell-inflamed immunosuppression from alternative immune-cold microenvironments characterized by low cytotoxic infiltration and non-canonical checkpoint patterns, adding further complexity to EMT-associated immune remodeling.

Based on these observations, the present study aimed to systematically evaluate the expression pattern and clinical significance of ZNF114 in gastric cancer and to elucidate its potential mechanisms of action. Through a series of *in vitro* and *in vivo* functional experiments, we investigated the effects of ZNF114 on gastric cancer cell proliferation, apoptosis, migration, and invasion, and further explored its association with Wnt/EMT signaling and the tumor immune microenvironment. This study is expected to provide new insights into the molecular mechanisms underlying gastric cancer progression and to offer a theoretical basis for the development of targeted therapeutic strategies.

Gastric cancer was prioritized in the present study because the strongest internally supported expression, prognostic, tissue-validation and functional evidence for ZNF114 was concentrated in this cancer type; the available in-house clinical specimens and functional models were gastric-cancer derived, and the pan-cancer analysis showed cancer-type-dependent differential expression rather than uniform dysregulation.

## 2 Materials and methods

### 2.1 Bioinformatics analysis

Transcriptomic expression profiles and clinical data for stomach adenocarcinoma (STAD) were obtained from The Cancer Genome Atlas (TCGA) (28), and the GEO datasets GSE79973, GSE65801, and GSE13861 were included as external validation cohorts. TIMER3.0 and Gene Expression Profiling Interactive Analysis (GEPIA) were used to characterize the pan-cancer expression pattern of ZNF114. According to the TIMER3.0 Gene_DE documentation, TCGA tumor-versus-adjacent-normal differential expression is analyzed using TCGA RNA-seq raw counts and edgeR. Because cancer-specific effect sizes and adjusted P values were not downloadable from the web interface, we additionally performed an independently defined reanalysis using the available expression matrix, two-sided Wilcoxon rank-sum tests and Benjamini–Hochberg correction. The association between ZNF114 and distinct tumor functional states at the single-cell level was assessed using the CancerSEA database (29). Gene set enrichment analysis (GSEA) (30) was performed to identify signaling pathways differentially enriched between the high- and low-ZNF114 expression groups, with particular attention to EMT and Wnt-related pathways. Gene sets were derived from the Molecular Signatures Database (MSigDB), and enrichment was considered significant at nominal P < 0.05 and false discovery rate (FDR) < 0.25. For TCGA-STAD expression-group comparisons, tumors were stratified into ZNF114-high and ZNF114-low groups using the median ZNF114 expression value as the cutoff. Immune-cell and pathway signature scores were calculated from TCGA-STAD transcriptomic data using predefined gene sets and z-score-based scoring. Single-sample gene set enrichment analysis (ssGSEA) was used to estimate the relative abundance of immune cell populations. Cytolytic activity was calculated as the mean z-score of granzyme A (GZMA) and perforin 1 (PRF1) expression across all tumors. Each immune program score (cytotoxic T/NK, T-cell-inflamed IFN, antigen presentation, DC/T-cell recruitment, stromal/myeloid suppression, Wnt/EMT) was calculated as the mean of gene-level z-scores, with each gene z-standardized across all TCGA-STAD tumors; all component genes were present in the expression matrix (**Supplementary Table S3**). The immune-cold index was then defined as z{mean(stromal/myeloid suppression, Wnt/EMT) − mean(cytotoxic T/NK, T-cell-inflamed IFN, antigen presentation, DC/T-cell recruitment)}, where the two programs on the suppressive side each contribute +1/2, the four immune-activation/recruitment programs each contribute −1/4, and z denotes re-standardization of the resulting difference across the cohort. No data-fitted feature weights or thresholds for classifying tumors by this index were applied; the index was analyzed as a continuous variable, whereas the ZNF114-high and ZNF114-low groups were defined by median ZNF114 expression. Checkpoint comparisons used two-sided Wilcoxon rank-sum tests, with Benjamini–Hochberg correction across the 11 checkpoint genes. The immune-cold index is a descriptive composite of these transcriptomic programs and has not been validated against an established immune-cold classification. For this signature analysis, Benjamini–Hochberg correction was applied across eight scores (the six component programs, cytolytic activity and the composite index), separately for group comparisons and correlations with ZNF114 expression. Checkpoint-gene expression, cytolytic activity, antigen presentation, T-cell-inflamed interferon (IFN) signatures, tumor mutational burden (TMB), genome-alteration features and microsatellite status were compared between ZNF114-high and ZNF114-low tumors. TMB was analyzed as mutations per megabase; total mutation count was treated as a separate genomic feature. Fraction genome altered and aneuploidy scores were derived from TCGA copy-number analysis results. Predicted drug sensitivity was estimated using the oncoPredict R package (31) (version 0.99) based on Genomics of Drug Sensitivity in Cancer (GDSC2) data, with TCGA-STAD transcriptomic profiles as input. Predicted half maximal inhibitory concentration (IC50) values were compared between ZNF114-high and ZNF114-low groups using the Wilcoxon rank-sum test, and results were treated as exploratory. For the TCGA-STAD analysis in **Figures 1A, B**, patients were ranked according to ZNF114 expression and divided into high- and low-expression groups using the median expression level as the cutoff. This was a single-gene expression-based stratification; no multivariable prognostic model was constructed for these panels. Receiver operating characteristic (ROC) analysis was performed using TCGA-STAD expression and clinical survival data to evaluate the prognostic discrimination of ZNF114 expression (**Figure 1C**). Separately, OS, FP, and PPS analysis in **Figures 1D–F** were generated using the gastric cancer module of Kaplan–Meier Plotter (https://kmplot.com/analysis/index.php?p=service&cancer=gastric), with probe set 1552946_at. Patient-selection settings were kept unchanged while the survival endpoint was switched. The baseline sample sizes were 631 for OS, 522 for FP, and 384 for PPS. These endpoint-specific analysis may include overlapping patients and were not treated as three independent validation cohorts. Candidate genes were prioritized based on both nominal prognostic significance and reproducibility across independent datasets. Because the goal was to identify a robust candidate for subsequent experimental validation, concordant cross-cohort evidence was considered more informative than a smaller P value observed in only one cohort.

**Figure 1:**
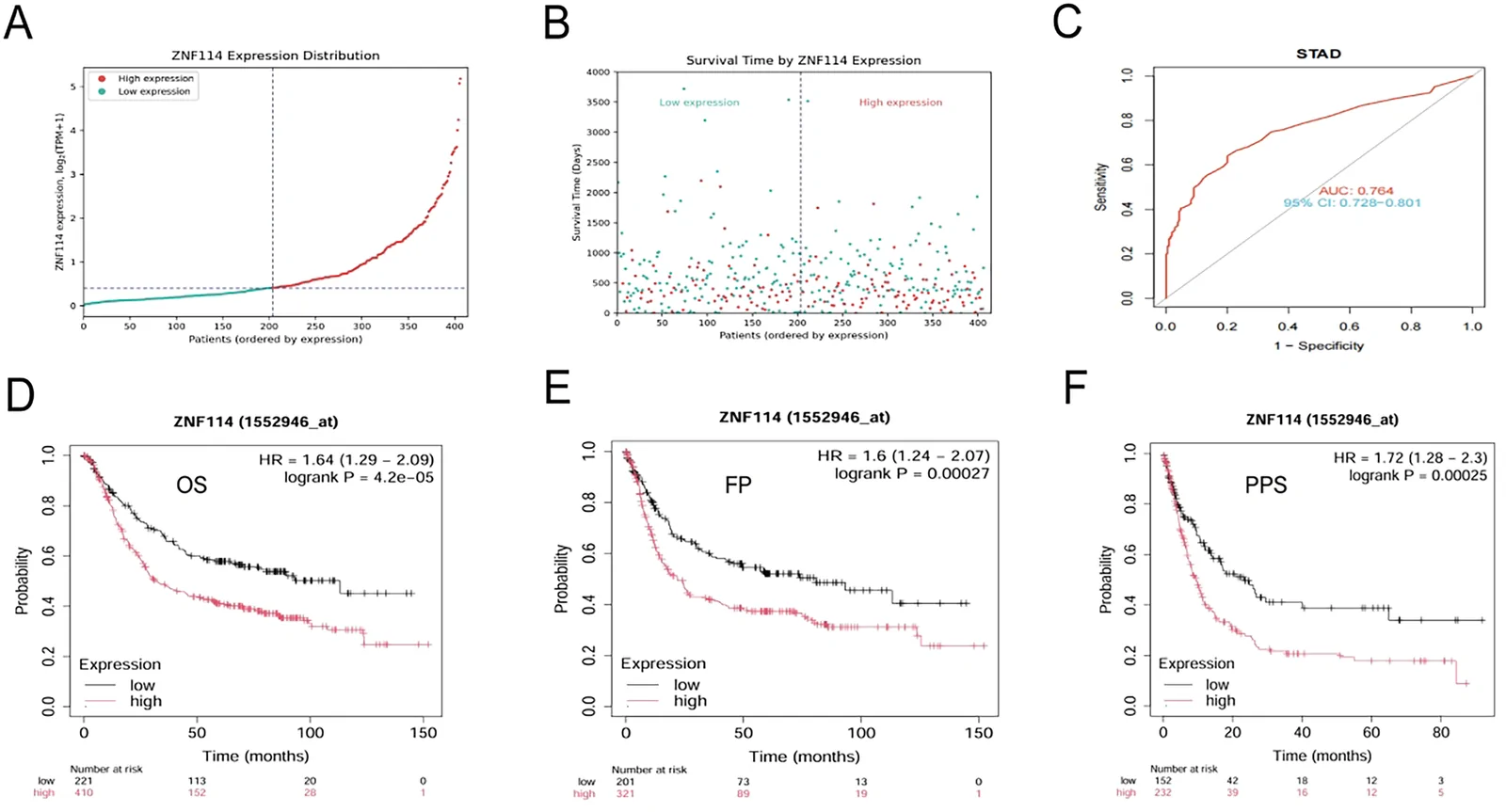
High ZNF114 expression is associated with poor prognosis in gastric cancer patients. **(A)** ZNF114 expression-based ordering of TCGA-STAD patients, stratified into high- and low-expression groups using the median ZNF114 expression level. **(B)** Survival time and status of TCGA-STAD patients ordered by ZNF114 expression. **(C)** Receiver operating characteristic (ROC) analysis of the prognostic discrimination of ZNF114 expression in TCGA-STAD (AUC = 0.764). **(D)** Kaplan-Meier survival curves showing that high ZNF114 expression is significantly associated with shorter overall survival (OS) (HR = 1.64, P = 4.2 × 10^-5^). **(E, F)** Kaplan-Meier curves demonstrating that high ZNF114 expression correlates with shorter first progression (FP) (HR = 1.60, P = 2.7 × 10^-4^) and post-progression survival (PPS) (HR = 1.72, P = 2.5 × 10^-4^). **(D–F)** Were generated using the gastric cancer module of Kaplan–Meier Plotter with probe set 1552946_at and unchanged patient-selection settings across endpoints. Baseline group sizes (low/high expression) were 221/410 for OS, 201/321 for FP, and 152/232 for PPS. P values are nominal log-rank P values without adjustment across the three survival endpoints. Patient sets may overlap; these panels are exploratory endpoint analysis, not three independent validation cohorts.

### 2.2 Human specimens

A total of six pairs of gastric cancer tissues and matched adjacent non-tumor tissues were collected from patients who underwent surgical resection at the Department of Gastrointestinal Surgery, Henan Provincial People’s Hospital, between January 1, 2023 and June 1, 2023. All cases were pathologically confirmed as gastric cancer. The specimens were immediately snap-frozen in liquid nitrogen after surgical resection and stored for subsequent analysis. None of the patients had received radiotherapy, chemotherapy, or immunotherapy prior to surgery. Written informed consent was obtained from all participants. The study protocol was approved by the Research Ethics Committee of Henan Provincial People’s Hospital. Corresponding tissue portions from the same six patient-matched pairs were used for Western blotting and immunohistochemical analysis. Thus, the in-house IHC cohort comprised six patients and 12 tissue specimens; it was not an independent tissue microarray cohort.

### 2.3 Cell lines and culture conditions

The human normal gastric mucosal epithelial cell line GES-1 and the gastric cancer cell lines MKN-45, AGS, and HGC-27 were purchased from Servicebio (Wuhan, China). Cells were cultured in Roswell Park Memorial Institute (RPMI)-1640 or Dulbecco’s Modified Eagle Medium (DMEM) supplemented with 10% fetal bovine serum (FBS) and 1% penicillin–streptomycin, and maintained at 37 °C in a humidified atmosphere containing 5% CO_2_.

### 2.4 siRNA transfection and establishment of stable knockdown cell lines

The three small interfering RNA (siRNA) sequences shown in **Figures 2A, B** targeting ZNF114, along with a negative control sequence (si-NC), were designed and synthesized by GenePharma (Shanghai, China). Transient transfection was carried out using Lipofectamine 3000 (Invitrogen) in accordance with the manufacturer’s protocol. At 48 h after transfection, total protein was extracted, and knockdown efficiency was evaluated by Western blotting. The siRNA with the highest silencing efficiency (si-2) was selected for subsequent experiments. For the *in vivo* assays, HGC-27 cells were infected with a lentiviral vector carrying ZNF114-targeting short hairpin RNA (shRNA). Following selection with 2 μg/mL puromycin for 2 weeks, stable ZNF114-knockdown cells (shZNF114) and corresponding control cells (shCTRL) were established. During establishment of the ZNF114 knockdown system, 12 ZNF114-targeting siRNA candidates obtained from three independent commercial sources were screened. si-2 produced the most consistent reduction in ZNF114 expression and was selected for subsequent phenotypic experiments; the other candidates showed insufficient or inconsistent knockdown for downstream assays. For rescue experiments in AGS and HGC-27 cells, the three groups were si-NC plus empty vector, si-2 plus empty vector, and si-2 plus the si-2-resistant ZNF114 construct. Cells were co-transfected with si-2 and an si-2-resistant ZNF114 rescue plasmid or the corresponding empty vector. The rescue construct contained synonymous substitutions within the si-2-targeted sequence without altering the encoded ZNF114 protein sequence.

**Figure 2:**
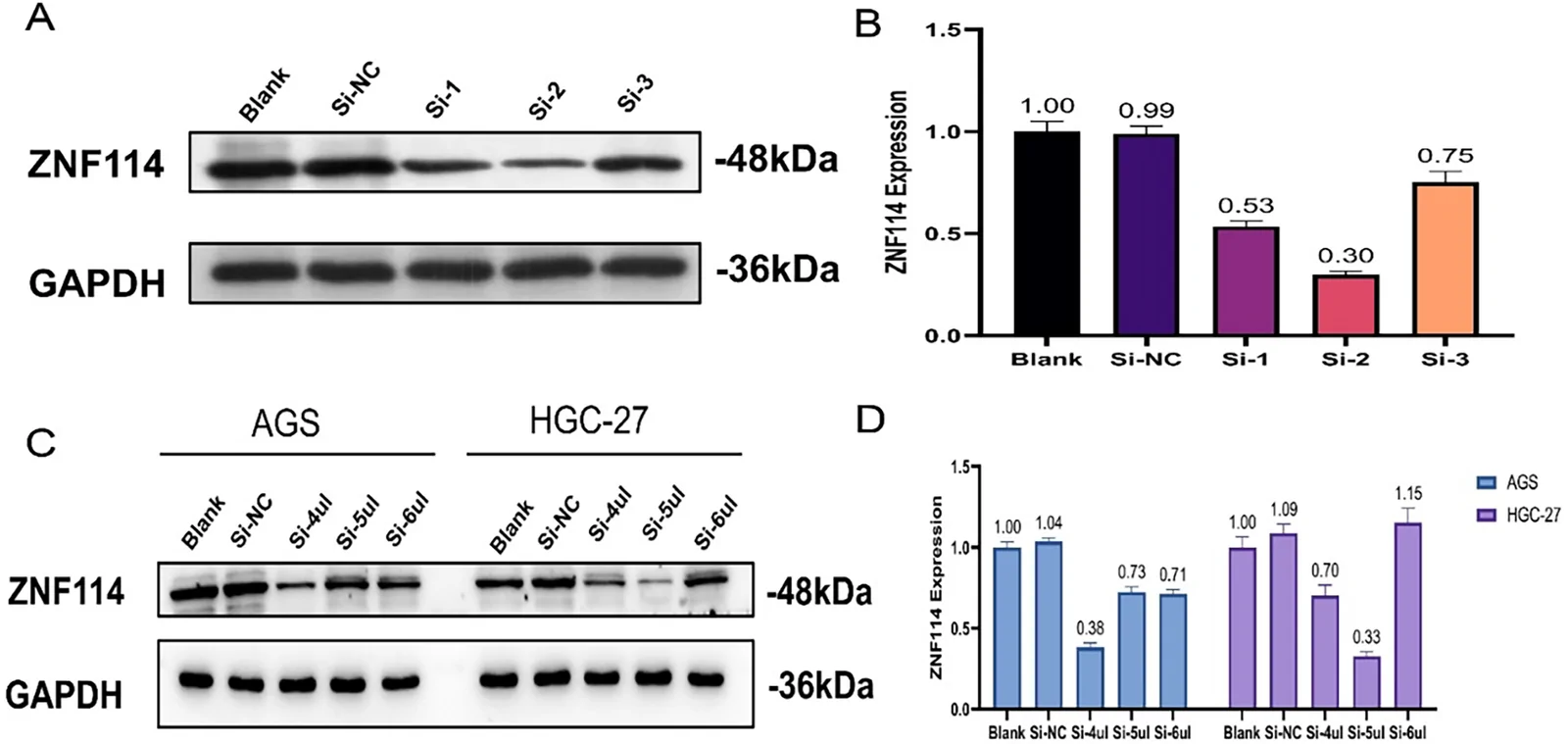
Establishment and validation of ZNF114-knockdown gastric cancer cell models. **(A, B)** Western blot analysis and quantification showing knockdown efficiency of three siRNA sequences (si-1, si-2, si-3) targeting ZNF114 in AGS cells, with si-2 demonstrating the highest silencing efficiency. **(C, D)** Dose optimization experiments showing ZNF114 protein expression in AGS and HGC-27 cells transfected with different volumes of si-2 siRNA. During assay optimization, 12 candidate siRNAs from three commercial sources were screened; si-2 was selected because it showed the most consistent silencing. Values are presented as normalized residual ZNF114 expression, with si-NC set to 100%; therefore, residual expression and knockdown efficiency should not be conflated.

### 2.5 Western blot

Total protein was extracted from cells or tissues using radioimmunoprecipitation assay (RIPA) lysis buffer containing protease inhibitors, and protein concentrations were measured using a bicinchoninic acid (BCA) protein assay kit. Equal amounts of protein (30 μg) were separated by 8% sodium dodecyl sulfate-polyacrylamide gel electrophoresis (SDS-PAGE) and transferred onto polyvinylidene fluoride (PVDF) membranes. After blocking with 5% nonfat milk for 2 h at room temperature, the membranes were incubated overnight at 4 °C with primary antibodies (all purchased from Servicebio, Wuhan, China). On the following day, the membranes were incubated with horseradish peroxidase (HRP)-conjugated secondary antibodies for 1 h at room temperature. Protein bands were detected using an enhanced chemiluminescence (ECL) reagent, and band intensities were quantified using ImageJ and Image Lab software.

### 2.6 CCK-8

Cell proliferation was evaluated using a Cell Counting Kit-8 (CCK-8; Servicebio). Transfected cells were seeded into 96-well plates at a density of 1 × 10³ cells per well. At 0, 24, 48, 72, and 96 h after seeding, 10 μL of CCK-8 solution was added to each well, and the cells were incubated at 37 °C for 2 h. Absorbance was measured at 450 nm using a microplate reader.

### 2.7 Colony formation

For the colony formation assay, cells were seeded into 6-well plates at a density of 500 cells per well and maintained under standard culture conditions for 14 days. The colonies were subsequently fixed with 4% paraformaldehyde, stained with crystal violet, and counted. Colonies consisting of at least 50 cells were considered viable clones.

### 2.8 Cell apoptosis assay

Cell apoptosis was evaluated using an Annexin V-fluorescein isothiocyanate (FITC)/propidium iodide (PI) Apoptosis Detection Kit (BD Biosciences). Briefly, logarithmically growing cells from each group were harvested, washed with PBS, and stained with Annexin V-FITC and propidium iodide (PI) in the dark according to the manufacturer’s protocol. Apoptotic cells were then analyzed using a BD FACSCanto II flow cytometer, and the overall apoptotic rate was determined by summing the percentages of early and late apoptotic cells.

### 2.9 Wound healing, cell migration and invasion assays

For the wound healing assay, cells were seeded into 6-well plates and grown to approximately 90% confluence. A linear scratch was created across the cell monolayer using a sterile 200-μL pipette tip, and detached cells were removed by washing with PBS. Wound closure was monitored and photographed at 0 h and 24 h under a microscope. The wound healing rate was calculated as the percentage of wound closure relative to the initial scratch area. Cell migration and invasion were evaluated using 8-μm Transwell chambers (Corning). For the migration assay, 2 × 10^4^ cells suspended in serum-free medium were added to the upper chamber. For the invasion assay, the upper chamber membrane was pre-coated with 50 μL of Matrigel (BD Biosciences; diluted 1:8) and allowed to polymerize before cell seeding. In both assays, complete medium containing 20% fetal bovine serum (FBS) was added to the lower chamber as a chemoattractant. After incubation for 24 h for migration or 48 h for invasion, cells remaining on the upper surface of the membrane were removed with cotton swabs. Cells that had migrated or invaded through the membrane were fixed with methanol, stained with crystal violet, and counted under a microscope in five randomly selected fields.

### 2.10 Immunohistochemical staining

Paraffin-embedded tissue sections were deparaffinized in xylene and rehydrated through a graded ethanol series. Antigen retrieval was performed in citrate buffer (pH 6.0) using heat-induced retrieval under high pressure. Endogenous peroxidase activity was blocked with 3% H_2_O_2_, and the sections were subsequently blocked with 5% bovine serum albumin (BSA) for 30 min at room temperature. The sections were then incubated overnight at 4 °C with primary antibodies against ZNF114 or Ki-67 (Servicebio, Wuhan, China). The next day, the sections were incubated with HRP-conjugated secondary antibodies, visualized with 3,3’-diaminobenzidine (DAB), counterstained with hematoxylin, dehydrated, and mounted with neutral resin. Stained sections were observed and photographed under a light microscope. The clinical tissue sections used for IHC were derived from the same six patient-matched pairs described in Section 2.2.

### 2.11 Subcutaneous xenograft assay in nude mice

Ten 7-week-old male BALB/c nude mice were purchased from Hunan SJA Laboratory Animal Co., Ltd. and randomly divided into the shCTRL group and the shZNF114 group (n = 5 per group). Stably transfected cells were resuspended in PBS, and 5 × 10^6^ cells in 100 μL were subcutaneously injected into the right axillary region of each mouse. Mice were anesthetized with isoflurane using a standard inhalation anesthesia system during the subcutaneous injection of cells. Anesthesia was induced with 3–4% isoflurane and maintained with 1–2% isoflurane in oxygen via a nose cone. The depth of anesthesia was monitored by respiratory rate and the absence of pedal withdrawal reflex. All efforts were made to minimize animal suffering. Beginning on day 7 after inoculation, tumor length (L) and width (W) were measured using calipers every 6 days (days 7, 13, 19, 25, and 31). Tumor volume was calculated according to the formula V = L × W²/2, and growth curves were plotted accordingly. On day 31, the mice were deeply anesthetized with isoflurane and then euthanized by cervical dislocation, after which the tumors were harvested and weighed. All animal procedures were performed following institutional guidelines for the care and use of laboratory animals and were approved by the Animal Ethics Committee of Henan Shuangyun Biotechnology Co., Ltd. (Approval No. SYLS2025131). All 10 animals were included in both the tumor-growth-curve analysis and the endpoint tumor-weight analysis; five animals were analyzed per group, and no animals were excluded.

### 2.12 DAB-TUNEL staining

TUNEL staining of xenograft paraffin sections was performed using an *in situ* apoptosis detection kit (DAB method). Briefly, after deparaffinization and rehydration, the sections were digested with proteinase K and incubated with the TUNEL reaction mixture at 37 °C for 60 min in the dark. The sections were subsequently incubated with converter-POD for 30 min at room temperature, visualized with DAB, counterstained with hematoxylin, and mounted. TUNEL-positive nuclei appeared brown. For quantitative analysis, five randomly selected high-power fields were evaluated for each section, and the percentage of TUNEL-positive cells was calculated.

### 2.13 Statistical analysis

*In vitro* experiments were repeated at least three times, and the data are presented as the mean ± standard deviation (SD). Statistical analysis and data visualization were performed using GraphPad Prism 9.0 and R software (version 4.4.1). The six patient-matched tissue pairs in **Figures 3H, I** were compared using the exact two-sided Wilcoxon matched-pairs signed-rank test. This single paired comparison was not subjected to high-throughput multiple-testing correction. Comparisons between two groups were performed using the independent-samples t-test for experimental assays or the two-sided Wilcoxon rank-sum test for non-normally distributed transcriptomic and predicted IC50 data. Comparisons among multiple groups were conducted using one-way analysis of variance (ANOVA) where appropriate. Survival curves were generated using the Kaplan-Meier method, and differences between groups were evaluated by the log-rank test. Correlations were assessed using Spearman correlation. P values from high-throughput feature comparisons were adjusted using the Benjamini-Hochberg false discovery rate (FDR) method. P < 0.05 was considered statistically significant. The log-rank P values reported for the Kaplan–Meier Plotter OS, FP, and PPS analysis are nominal and were not adjusted for testing multiple survival endpoints; these analysis were interpreted as related exploratory findings.

**Figure 3:**
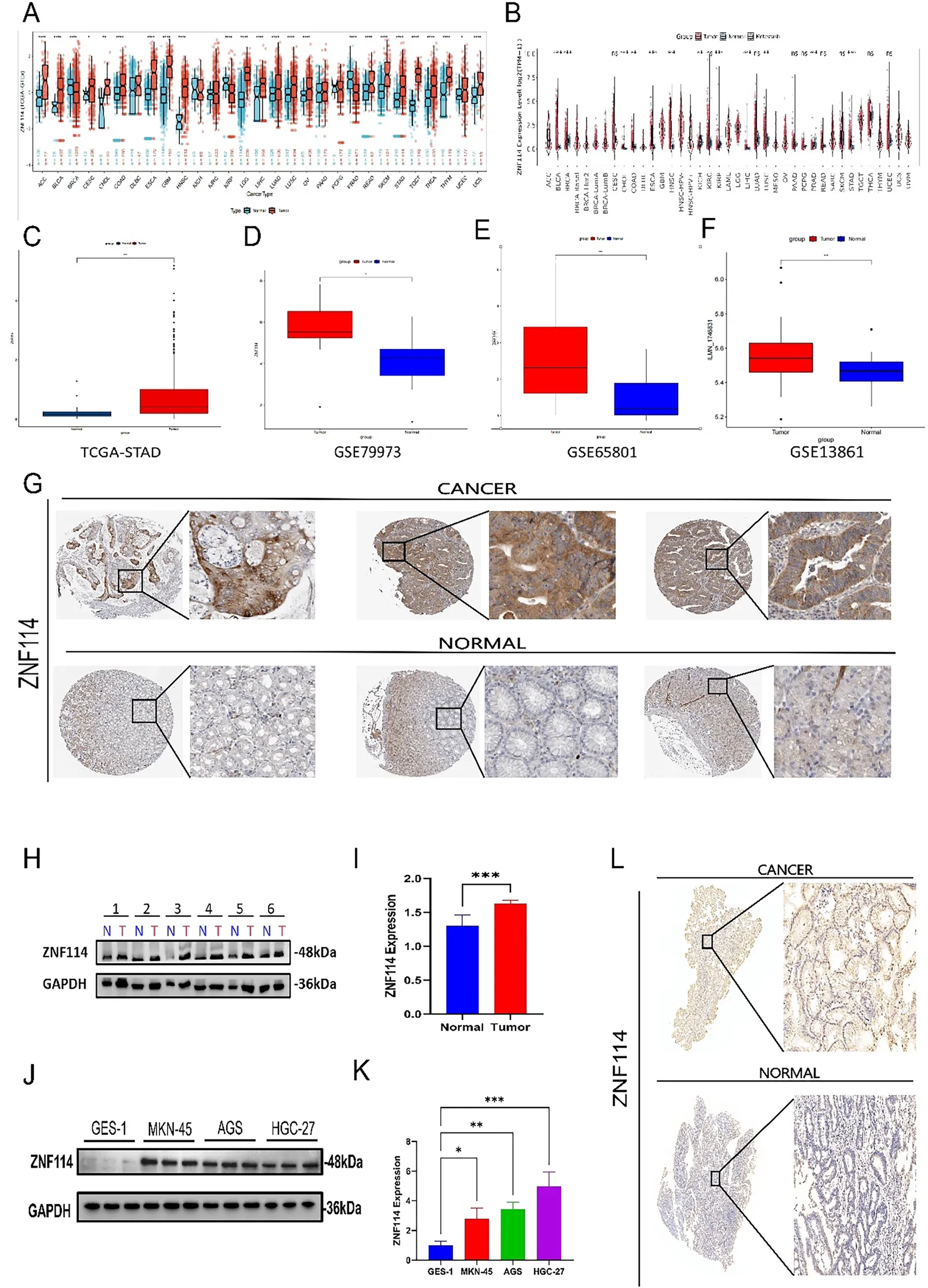
ZNF114 is significantly upregulated in gastric cancer tissues and cell lines. **(A, B)** Pan-cancer expression analysis of ZNF114 using TIMER3.0 and GEPIA, showing cancer-type-dependent differential expression, including significant upregulation in multiple cancer types and in stomach adenocarcinoma (STAD), but not uniform dysregulation across all cancer types. **(C–F)** ZNF114 mRNA expression in gastric cancer tissues compared with normal tissues across TCGA-STAD and GEO datasets. **(G)** Representative immunohistochemical images from the Human Protein Atlas showing ZNF114 protein expression in gastric cancer tissues. **(H, I)** Western blot analysis and quantification of ZNF114 protein expression in six patient-matched pairs of gastric cancer and adjacent non-tumor tissues (exact two-sided Wilcoxon matched-pairs signed-rank P = 0.03125; *P < 0.05). **(J, K)** Western blot analysis and quantification of ZNF114 protein expression in normal gastric epithelial cell line GES-1 and gastric cancer cell lines MKN-45, AGS, and HGC-27. **(L)** Representative immunohistochemical staining of ZNF114 in the same six paired clinical gastric cancer and adjacent non-tumor tissue specimens used for the Western blot analysis. This panel represents the same six-patient, 12-specimen in-house cohort and not an independent tissue microarray.

## 3 Results

### 3.1 ZNF114 is significantly upregulated in gastric cancer tissues and cells

To prioritize a C2H2-type zinc finger candidate relevant to gastric cancer progression, we performed an integrated screening workflow across expression, prognosis and functional-state analysis, which identified ZNF114 as a candidate for further validation (**Supplementary Figure S1**). The pan-cancer expression pattern of ZNF114 was analyzed using the TIMER3.0 and GEPIA databases. The results showed cancer-type-dependent differential expression. In the TCGA–GTEx reanalysis corresponding to **Figure 3A**, ZNF114 was significantly upregulated in 21 of 29 evaluated cancer types, significantly downregulated in 3 cancer types, and not significantly different in 5 cancer types after Benjamini–Hochberg correction; STAD was among the upregulated cancer types. The independent TCGA tumor-versus-adjacent-normal reanalysis corresponding to **Figure 3B** showed concordant upregulation in STAD (**Supplementary Tables S1**, **S2**). In STAD, the TCGA–GTEx analysis included 414 tumor samples, 36 TCGA adjacent-normal samples, and 174 GTEx normal samples; the median expression difference on the supplied transformed-expression scale was 1.9787 (two-sided Wilcoxon P = 3.54 × 10^-27; BH-adjusted P = 1.71 × 10^-26). The independent TCGA tumor-versus-adjacent-normal reanalysis included 414 tumor samples and 36 adjacent-normal samples, with a median difference of 1.8495 on the same transformed-expression scale (two-sided Wilcoxon P = 4.73 × 10^-6; BH-adjusted P = 1.66 × 10^-5). Integrated analysis of the TCGA-STAD cohort and three external GEO datasets (GSE79973, GSE65801, and GSE13861) further confirmed the upregulation of ZNF114 in gastric cancer tissues compared with normal tissues (**Figures 3C–F**). Western blotting showed consistently higher ZNF114 protein expression in gastric cancer tissues than in the matched adjacent non-tumor tissues across all six pairs (two-sided Wilcoxon matched-pairs signed-rank test, exact P = 0.03125; **Figures 3H, I**). In-house immunohistochemistry provided complementary protein-level support in the same six paired clinical specimens (**Figure 3L**). Given the small sample size (n = 6 paired specimens), these protein-level findings should be regarded as preliminary. ZNF114 was also elevated in gastric cancer cell lines compared with the normal gastric epithelial cell line GES-1 (**Figures 3J, K**). These data indicate that ZNF114 is aberrantly upregulated in gastric cancer. Although ZNF521 showed a stronger nominal Cox P value in the initial cohort, its cross-cohort expression support was less consistent. In contrast, ZNF114 showed concordant support across all three GEO datasets and was therefore prioritized for subsequent validation.

### 3.2 High ZNF114 expression is associated with poor prognosis in patients with gastric cancer

Patients in the TCGA-STAD cohort were stratified into high- and low-ZNF114 expression groups using the median expression level. The expression distribution and corresponding survival status were visualized with patients ordered by ZNF114 expression (**Figures 1A, B**). ROC curve analysis showed that ZNF114 achieved an area under the curve (AUC) of 0.764 for prognostic discrimination in the TCGA-STAD cohort (**Figure 1C**). Separate Kaplan–Meier Plotter analysis showed that patients with high ZNF114 expression had significantly shorter overall survival (OS) than those with low ZNF114 expression (hazard ratio (HR) = 1.64, P = 4.2 × 10^-5^; **Figure 1D**). Moreover, high ZNF114 expression was also significantly associated with shorter first progression (FP) (HR = 1.60, P = 2.7 × 10^-4^) and post-progression survival (PPS) (HR = 1.72, P = 2.5 × 10^-4^) (**Figures 1E, F**). The OS, FP, and PPS findings represent related exploratory associations across different endpoints, rather than three independent confirmations of prognostic performance.

### 3.3 Establishment of ZNF114-knockdown cell lines

To evaluate the biological role of ZNF114 in gastric cancer, siRNA-mediated knockdown models were established. Western blot analysis showed that, compared with the si-NC group, the si-2 sequence achieved the most effective suppression of ZNF114 protein expression in AGS cells (**Figures 2A, B**). Further dose optimization experiments demonstrated that 4 μL and 5 μL of siRNA yielded the optimal silencing efficiency in AGS and HGC-27 cells, respectively (**Figures 2C, D**). Accordingly, the si-2 sequence together with the optimized transfection doses was used in subsequent experiments. In the original normalization, si-NC was set to 100%. Thus, the approximately 38% value in AGS cells treated with 4 μL si-2 represents residual ZNF114 expression and corresponds to an estimated knockdown efficiency of approximately 62%. In HGC-27 cells, the selected intermediate si-2 condition produced an approximately 70–73% reduction, whereas the highest tested dose caused an apparent rebound above the si-NC level and was not used for subsequent functional assays. The selected doses were based on the reproducible directional reduction in ZNF114 expression and acceptable experimental performance, rather than simply the highest tested dose.

### 3.4 ZNF114 promotes gastric cancer cell proliferation and inhibits apoptosis

CCK-8 assays showed that, compared with the control group, the viability of AGS and HGC-27 cells in the ZNF114-knockdown group was significantly reduced in a time-dependent manner (**Figures 4A, B**). Consistently, colony formation assays demonstrated that ZNF114 silencing markedly decreased the number of colonies formed (**Figures 4E, F**). Flow cytometric analysis further revealed that the apoptotic rate was significantly increased in the ZNF114-knockdown group compared with the si-NC group (**Figures 4G, H**). In addition, Western blot analysis showed that ZNF114 knockdown upregulated the expression of the pro-apoptotic protein Bax and downregulated the expression of the anti-apoptotic protein Bcl-2 (**Figure 4C**). Quantitative analysis of the Bcl-2/Bax ratio across three experimental replicates further supported a shift toward a pro-apoptotic protein balance following ZNF114 knockdown (**Figure 4D**).

**Figure 4:**
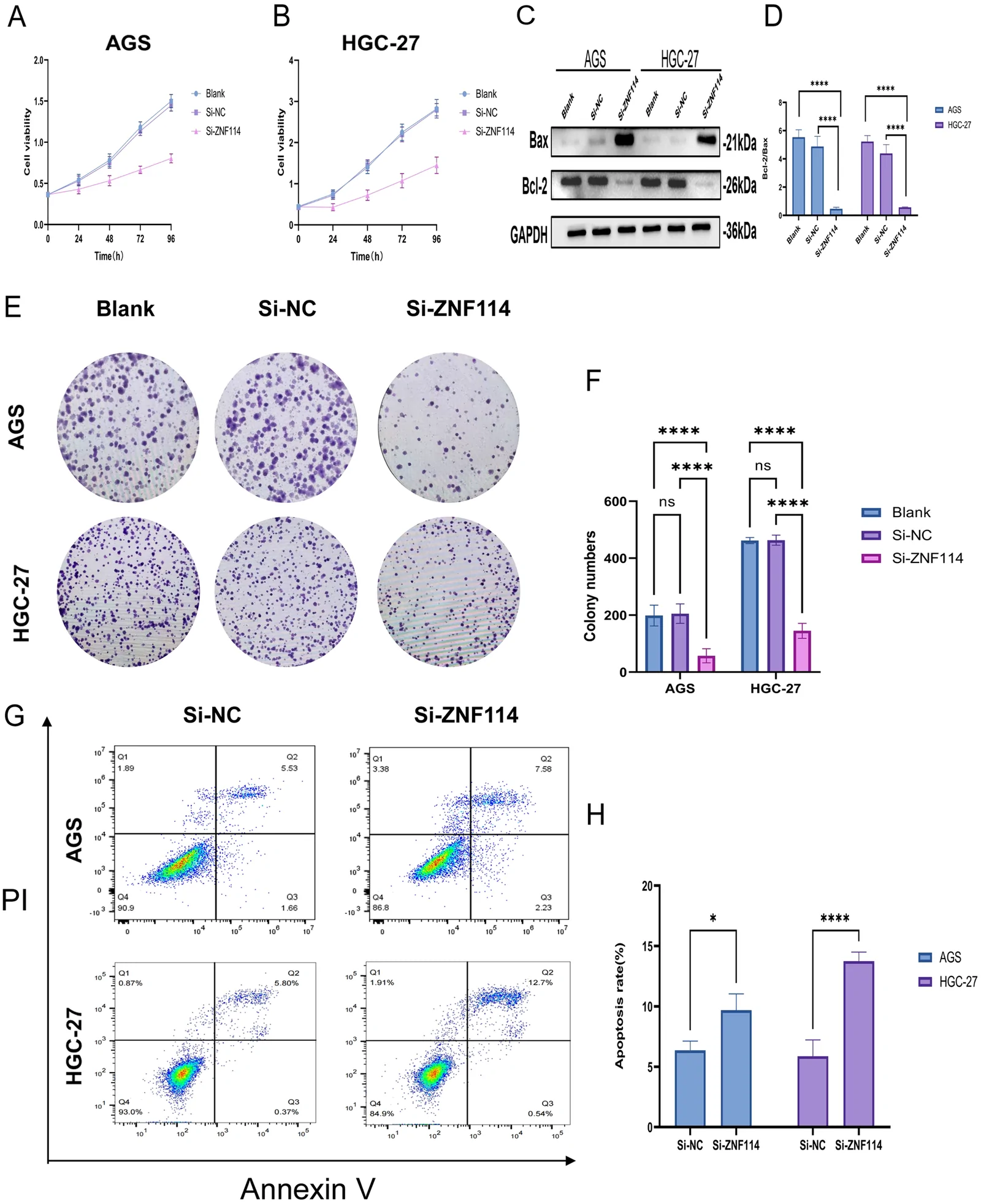
ZNF114 knockdown inhibits gastric cancer cell proliferation and promotes apoptosis. **(A, B)** CCK-8 assays showing that ZNF114 silencing significantly suppresses cell viability in AGS and HGC-27 cells at 24, 48, 72, and 96 h post-transfection. **(C)** Western blot analysis showing that ZNF114 knockdown upregulates the expression of the pro-apoptotic protein Bax and downregulates the expression of the anti-apoptotic protein Bcl-2 in AGS and HGC-27 cells. **(D)** Quantification of the Bcl-2/Bax ratio was based on densitometric measurements from three experimental replicates (n = 3); the underlying raw measurements and calculated ratios are provided in the source-data file. **(E, F)** Colony formation assays demonstrating that ZNF114 knockdown markedly reduces the colony-forming capacity of AGS and HGC-27 cells. **(G, H)** Flow cytometry analysis and quantification of apoptosis showing that ZNF114 silencing significantly increases the apoptotic rate in both cell lines.

### 3.5 ZNF114 promotes gastric cancer cell migration and invasion in association with Wnt and EMT changes

Wound healing assays showed that, compared with the blank and si-NC groups, AGS and HGC-27 cells in the si-ZNF114 group displayed significantly delayed wound closure at 24 h, accompanied by a reduced wound healing rate, suggesting impaired migratory ability after ZNF114 knockdown (**Figures 5A, B**). Consistent with this finding, Transwell migration and invasion assays showed that ZNF114 silencing markedly reduced the number of migrated and invaded gastric cancer cells (**Figures 5C–F**). CancerSEA analysis linked ZNF114 expression to EMT and invasion-related functional states. GSEA supported EMT enrichment, whereas Wnt/β-catenin enrichment did not meet the stated significance criteria (**Figure 5H**). Western blotting in both AGS and HGC-27 cells confirmed that ZNF114 knockdown increased E-cadherin and decreased N-cadherin, Vimentin and the Wnt-related proteins β-catenin, c-Myc, Cyclin D1 and p-GSK3β (**Figures 5I, J**). These results indicate that ZNF114 promotes gastric cancer cell migration and invasion in association with Wnt/EMT activation. To further assess the specificity of the si-2-mediated effects, rescue experiments were performed in AGS and HGC-27 cells (**Supplementary Figure S2**). Compared with the si-2 plus empty-vector group, re-expression of ZNF114 attenuated the EMT-associated protein changes and partially restored the impaired colony formation, wound-closure, migration and invasion phenotypes. These findings provide complementary support for the specificity of the ZNF114 knockdown-related phenotypes.

**Figure 5:**
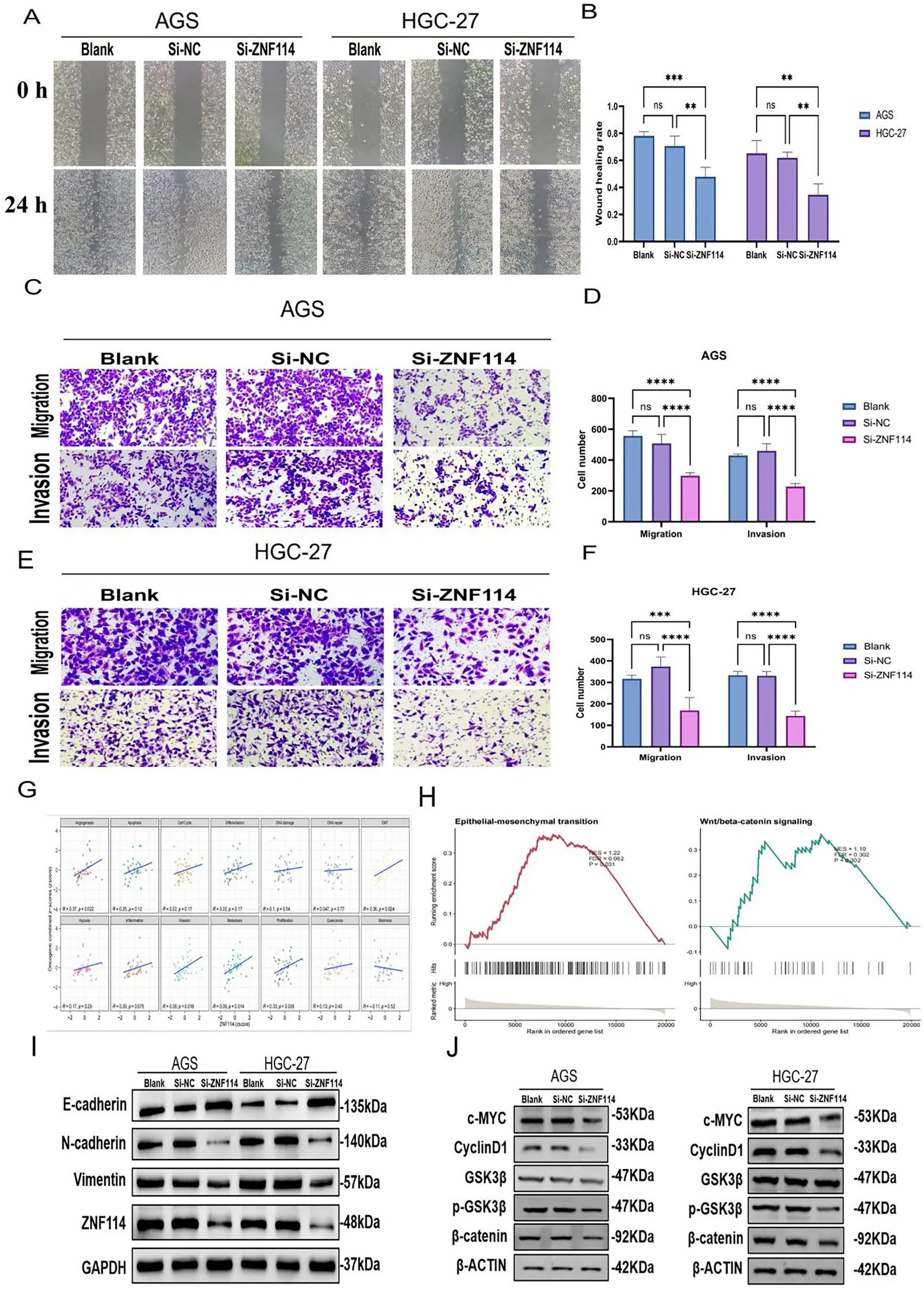
ZNF114 knockdown suppresses gastric cancer cell migration and invasion and is accompanied by Wnt/EMT-related protein changes. **(A, B)** Wound healing assays showing that ZNF114 knockdown delays wound closure in AGS and HGC-27 cells. **(C, D)** Transwell migration and invasion assays and quantification in AGS cells. **(E, F)** Corresponding migration and invasion assays and quantification in HGC-27 cells. **(G)** CancerSEA analysis showing positive correlation between ZNF114 expression and EMT/invasion functional states. **(H)** GSEA plots for EMT and Wnt/β-catenin signaling in the high-ZNF114 expression group. EMT met the stated enrichment criteria; Wnt/β-catenin did not (NES = 1.10, nominal P = 0.302, FDR = 0.302). **(I)** Western blot validation showing that ZNF114 knockdown upregulates E-cadherin and downregulates N-cadherin and Vimentin. **(J)** Western blot validation in AGS and HGC-27 cells showing that ZNF114 knockdown decreases Wnt-pathway proteins including beta-catenin, c-Myc, Cyclin D1, and p-GSK3beta, with total GSK3β also assessed and β-actin used as the loading control. Rescue experiments using an si-2-resistant ZNF114 construct in AGS and HGC-27 cells provided complementary specificity validation (**Supplementary Figure S2**).

Rescue-experiment data are shown in **Supplementary Figure S2**.

### 3.6 ZNF114 knockdown inhibits tumor growth *in vivo*

To further validate the tumor-promoting role of ZNF114 *in vivo*, a subcutaneous xenograft model was established using HGC-27 cells with stable ZNF114 knockdown. Compared with the shCTRL group, tumors in the shZNF114 group exhibited a markedly slower growth rate (**Figures 6A, B**). Consistently, final tumor weight was significantly lower in the shZNF114 group than in the shCTRL group (**Figure 6C**). Histological analysis further corroborated these data. Ki-67 immunohistochemical staining showed a significant reduction in the proportion of Ki-67-positive cells in the shZNF114 group, indicating decreased tumor cell proliferative activity (**Figures 6D–F**). In addition, DAB-TUNEL staining demonstrated that TUNEL-positive signals were localized in the nuclei and appeared brown. Compared with the shCTRL group, tumors in the shZNF114 group showed a significantly increased number of TUNEL-positive cells, suggesting enhanced apoptosis (**Figures 6G–I**). These *in vivo* results were consistent with the *in vitro* findings and further confirmed that ZNF114 promotes gastric cancer growth while inhibiting apoptosis. The experiment included five mice in each group for both the longitudinal tumor-growth analysis and the endpoint tumor-weight comparison; all animals were retained and no exclusions were made.

**Figure 6:**
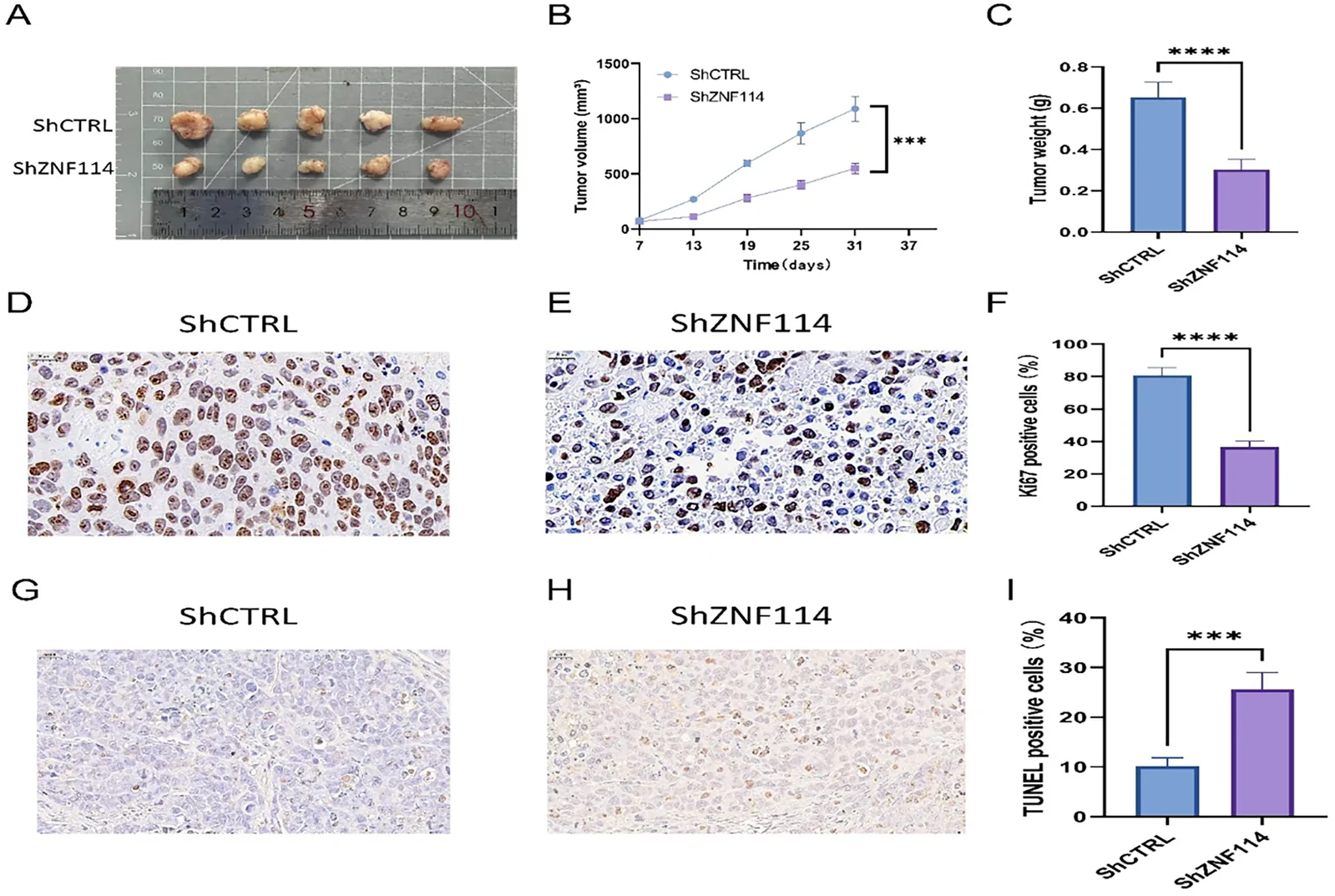
ZNF114 knockdown inhibits tumor growth *in vivo*. **(A, B)** Representative images and growth curves of subcutaneous xenograft tumors in nude mice showing that the shZNF114 group exhibits significantly slower tumor growth compared with the shCTRL group. **(C)** Final tumor weight showing that the shZNF114 group has significantly lower tumor weight than the shCTRL group. **(D–F)** Ki-67 immunohistochemical staining and quantification showing that the proportion of Ki-67-positive cells is significantly reduced in the shZNF114 group compared with the shCTRL group. **(G–I)** DAB-TUNEL staining and quantification showing that TUNEL-positive cells are significantly increased in the shZNF114 group compared with the shCTRL group, indicating enhanced apoptosis *in vivo*. Tumor-growth curves and endpoint tumor-weight comparisons were based on n = 5 mice per group, with no exclusions.

### 3.7 ZNF114-high tumors show elevated Wnt/EMT scores and a higher composite immune-cold index

To further investigate whether ZNF114-associated Wnt/EMT activation was linked to immune-related transcriptomic features, 410 TCGA-STAD tumors were stratified by median ZNF114 expression into high- and low-expression groups (n = 205 each). ZNF114-high tumors showed significantly elevated Wnt/EMT program scores (FDR < 0.001) and a higher immune-cold index (FDR < 0.001) (**Figure 7A**). Among the individual features, stromal/myeloid suppression was significantly higher in ZNF114-high tumors (FDR = 0.002), whereas cytotoxic T/NK (FDR = 0.252), T-cell-inflamed IFN (FDR = 0.327) and antigen presentation (FDR = 0.115) showed numerically lower scores without statistical significance after FDR correction. DC/T-cell recruitment showed a near-zero median difference and was also non-significant (FDR = 0.707). Spearman analysis across all tumors showed a similar pattern: ZNF114 expression was associated with stromal/myeloid suppression (ρ = 0.15, FDR = 0.006) and lower antigen presentation (ρ = −0.14, FDR = 0.010), while cytotoxic T/NK (ρ = −0.10, FDR = 0.070) and T-cell-inflamed IFN (ρ = −0.08, FDR = 0.140) showed weaker, non-significant trends (**Figures 7B, C**). The Wnt/EMT program score showed a moderate positive correlation with the immune-cold index (ρ = 0.53, P < 0.001) (**Figure 7D**). Because Wnt/EMT contributes to the composite index, the **Figure 7D** association is interpreted as exploratory and is not considered an independent validation. Thus, the most robust associations were observed for the Wnt/EMT program and the composite immune-cold index, whereas the cytotoxic T/NK, T-cell-inflamed IFN and antigen-presentation features showed non-significant downward trends. The exact BH-adjusted FDR for the composite immune-cold index was 5.5 × 10−6.

**Figure 7:**
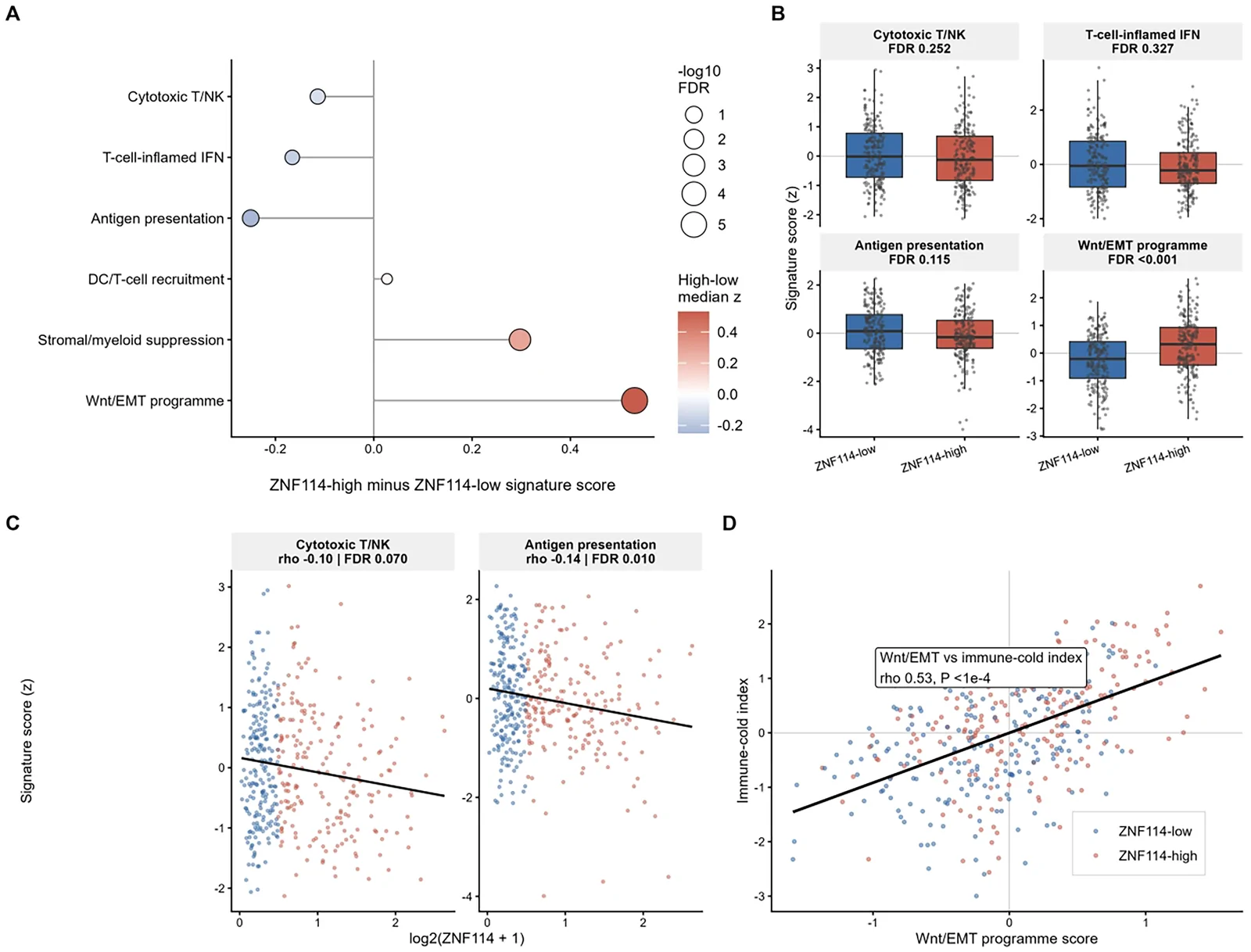
High ZNF114 expression is associated with Wnt/EMT and immune-related transcriptomic features in TCGA-STAD. **(A)** High-low median z-score differences across immune and Wnt/EMT-related signatures in TCGA-STAD (410 tumors; n = 205 in each ZNF114 expression group). Point color denotes direction and point size denotes -log10 FDR. Near-zero estimates close to the reference line, such as DC/T-cell recruitment, indicate minimal group difference rather than missing data. **(B)** Representative signature score distributions between ZNF114-high and ZNF114-low tumors. **(C)** Continuous associations between ZNF114 expression and selected immune activation signatures. **(D)** Moderate positive correlation between Wnt/EMT program score and the immune-cold index (Spearman ρ = 0.53). Because Wnt/EMT contributes to the index, this association is exploratory and does not provide independent validation.

### 3.8 ZNF114-high tumors exhibit altered checkpoint landscape and lower tumor mutational burden

We then examined whether this transcriptomic pattern was accompanied by a distinct immune-checkpoint and immunogenicity landscape. ZNF114-high tumors did not display a classical PDCD1/CD274/CTLA4-high T-cell-inflamed checkpoint pattern. Instead, alternative suppressive axes including CD276/B7-H3 and ADORA2A were significantly increased, whereas LGALS9 was reduced (**Figures 8A, B**). Integrated visualization summarized checkpoint features, genomic features, and immune-activation scores across ZNF114 expression groups (**Figure 8C**). ZNF114-high tumors also exhibited lower TMB and higher genome-alteration and aneuploidy features (**Figure 8D**). Together, the above data suggest that ZNF114-high gastric cancers are associated with elevated Wnt/EMT scores and altered immune-related transcriptomic features, including non-classical checkpoint differences, although the functional implications require further investigation. CTLA4 showed a small numerical increase without a statistically significant group difference (high-minus-low median difference = 0.1173 on the analyzed expression scale, or 0.1108 on the z-score scale; two-sided Wilcoxon P = 0.734427; BH-adjusted FDR = 0.807059; n = 205 per group). These findings do not support coordinated elevation of PDCD1, CD274 and CTLA4, but non-significance does not establish biological equivalence (**Supplementary Table S4**).

**Figure 8:**
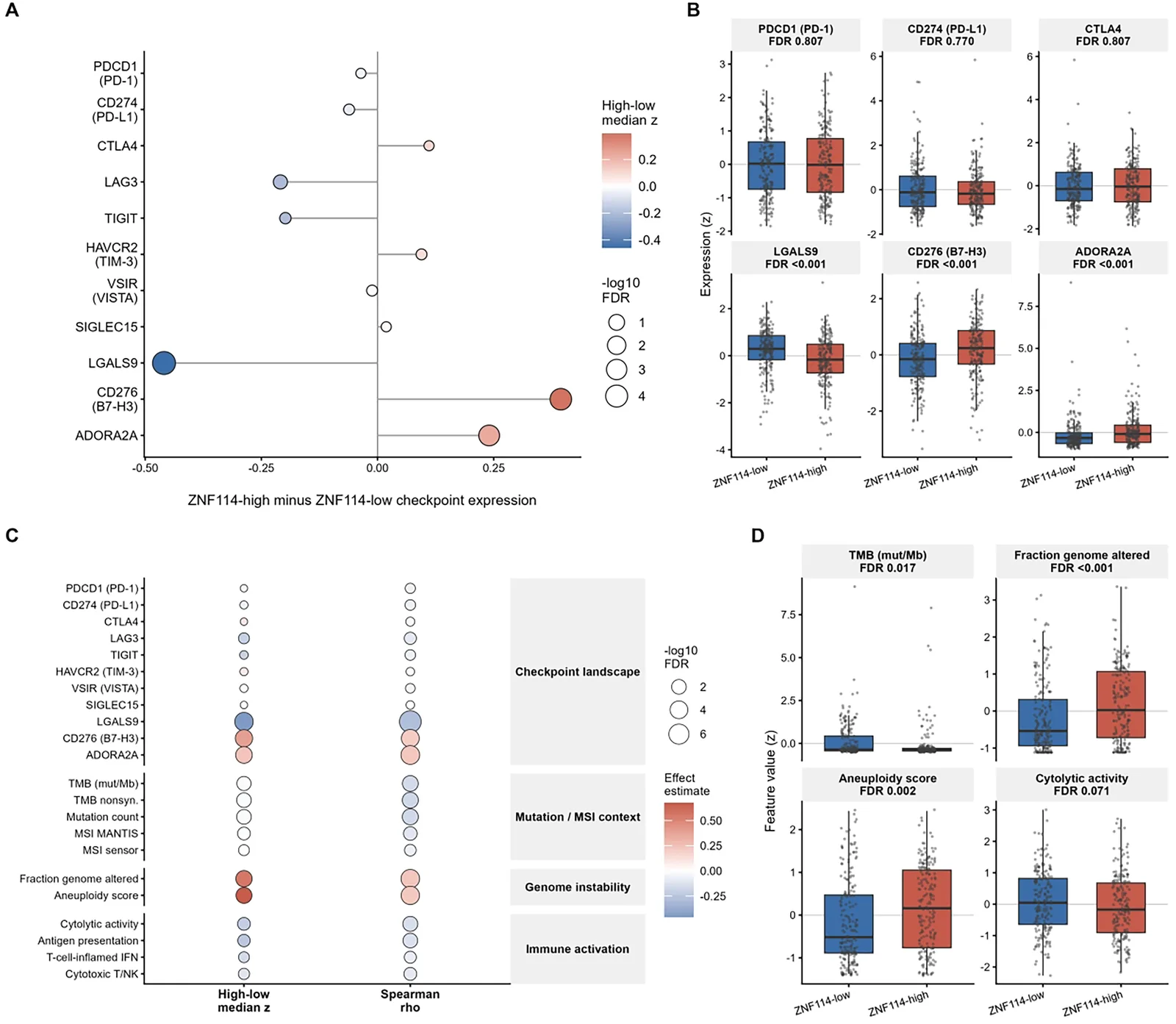
ZNF114-high tumors exhibit checkpoint alteration and lower tumor mutational burden. **(A)** High-low differences in immune checkpoint expression. Classical PDCD1/CD274/CTLA4-high inflammation was not the dominant pattern, whereas CD276/B7-H3 and ADORA2A were increased and LGALS9 was decreased in ZNF114-high tumors. Near-zero estimates close to the reference line, such as VSIR, indicate minimal group difference rather than missing data. **(B)** Expression distributions of representative checkpoint genes. **(C)** Integrated dot heatmap summarizing group differences and continuous ZNF114 associations across checkpoint, genomic and immune-activation features. **(D)** Distributions of representative immunogenic features, including TMB, fraction genome altered, aneuploidy score and cytolytic activity. CTLA4 showed a small numerical increase without statistical significance (high-minus-low median z-score difference = 0.1108; P = 0.734427; BH-adjusted FDR = 0.807059; n = 205 per group). The data do not support a coordinated PDCD1/CD274/CTLA4-high state (**Supplementary Table S4**).

### 3.9 Exploratory drug-sensitivity analysis reveals predicted IC50 differences associated with ZNF114 expression

Given the association between ZNF114 expression, Wnt/EMT activation, and lower tumor mutational burden, we explored whether ZNF114 expression was associated with predicted therapeutic sensitivity. Using the oncoPredict algorithm based on GDSC2 data, predicted IC50 values were compared between ZNF114-high and ZNF114-low tumors. Several gastric cancer chemotherapeutic agents, including oxaliplatin, 5-fluorouracil, irinotecan, and cisplatin, showed higher predicted IC50 values in ZNF114-high tumors, suggesting potentially reduced sensitivity (**Figures 9A–C**).

**Figure 9:**
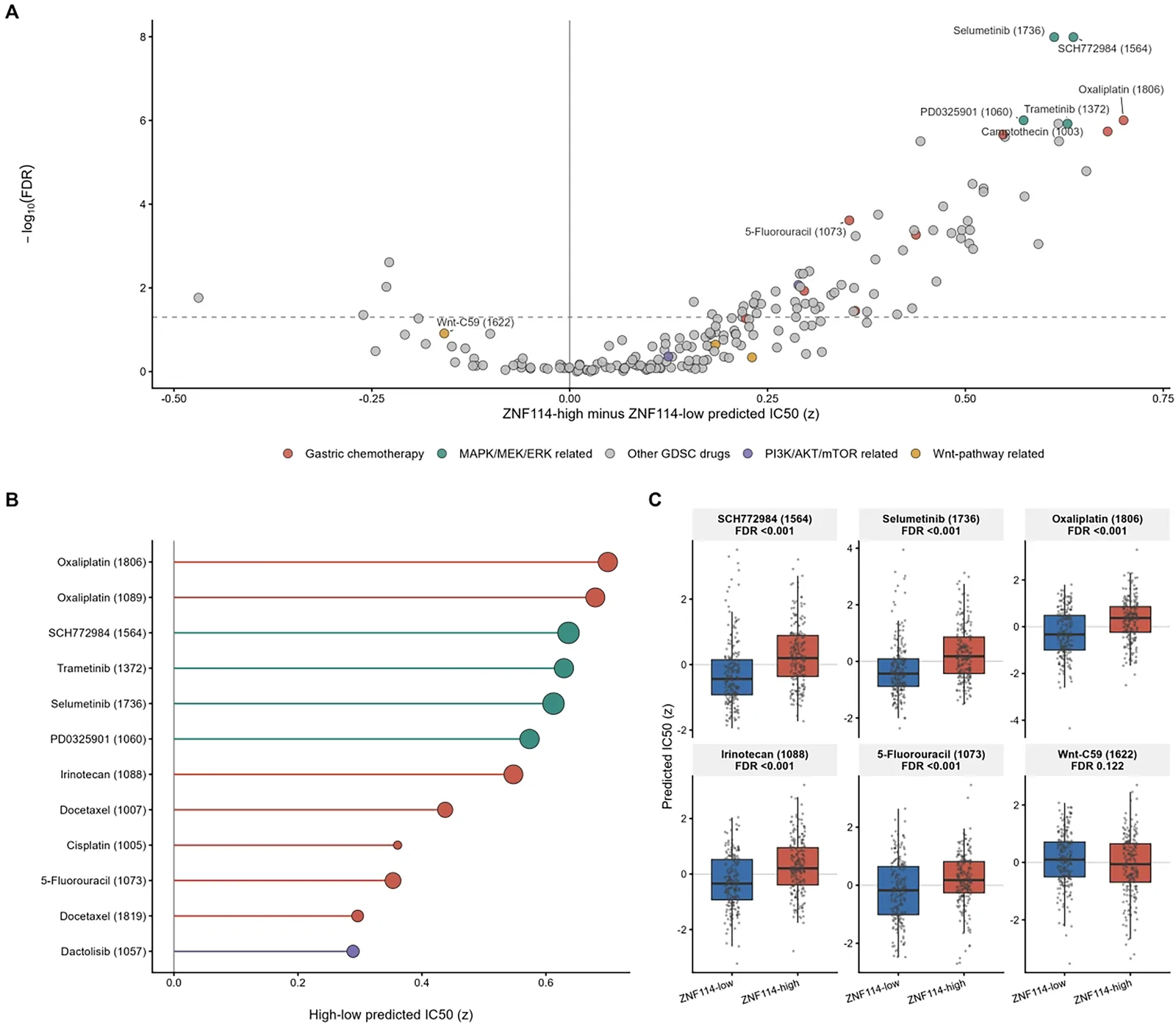
ZNF114 expression is associated with predicted drug-sensitivity differences. **(A)** Volcano plot of high-low predicted IC50 differences across compounds. Positive values indicate higher predicted IC50 and lower predicted sensitivity in ZNF114-high tumors. **(B)** Priority drug lollipop plot showing gastric chemotherapy and pathway-related compounds. **(C)** Predicted IC50 distributions for representative compounds. These analyses are computational and hypothesis-generating.

## 4 Discussion

In the present study, we systematically investigated the expression pattern, clinical significance and biological role of ZNF114 in gastric cancer. ZNF114 was markedly upregulated in gastric cancer tissues and cell lines, and high expression was significantly associated with unfavorable prognosis. Functional experiments demonstrated that ZNF114 silencing suppressed gastric cancer cell proliferation, migration and invasion, promoted apoptosis and inhibited tumor growth *in vivo*. ZNF114 knockdown was accompanied by changes in Wnt/EMT-related proteins, and high ZNF114 expression was associated with a higher composite immune-cold index in TCGA-STAD. These findings support a contribution of ZNF114 to malignant phenotypes in gastric cancer; the immune-related associations provide hypotheses for subsequent investigation. The integrated screening strategy (**Supplementary Figure S1**) provided a transparent rationale for prioritizing ZNF114 among C2H2-type zinc finger candidates through combined expression, prognostic and functional-state analysis.

Prior reports have suggested that ZNF114 is differentially expressed in selected malignancies and may be associated with unfavorable clinical outcomes. For example, ZNF114 has been shown to be significantly upregulated in oral cancer, and its high expression correlates with worse overall survival (15). In prostate cancer, ZNF114 was included in an exosome-related gene signature for tumor status classification and prognostic assessment (16). In our pan-cancer analysis, ZNF114 showed a cancer-type-dependent expression pattern rather than uniform dysregulation across all evaluated cancer types. The strongest internally supported expression, prognostic, tissue-validation and functional evidence in the present study was obtained in gastric cancer. Therefore, the present findings support a gastric-cancer-associated role for ZNF114, while its relevance in other tumor types remains to be determined. ZNF114 belongs to the C2H2-type zinc finger protein family, whose members are broadly involved in tumor development and progression and play critical roles in transcriptional regulation, cell survival, migration and metastasis, and remodeling of the tumor microenvironment (3, 4). For instance, ZNF395 is highly expressed in glioma and is associated with alterations in the immune microenvironment (17). ZNF827 is strongly induced during breast cancer metastasis and brain development-related EMT, implying a potential role in tumor dissemination (18). ZNF655 is markedly upregulated in ovarian cancer, and its knockdown inhibits tumor cell proliferation and migration (19). By contrast, ZNF133 and ZNF652 appear to exert tumor-suppressive effects, with their reduced expression being associated with tumor progression and poor prognosis (20, 21). These studies collectively underscore the functional heterogeneity of zinc finger protein family members in cancer. Compared with other family members, previous research on ZNF114 has remained largely confined to bioinformatics screening, with limited functional validation. Here, we extend the current understanding of ZNF114 by providing experimental evidence, both *in vitro* and *in vivo*, supporting its functional role in gastric cancer.

Aberrant activation of epithelial-mesenchymal transition (EMT) and Wnt signaling is a critical molecular basis for gastric cancer invasion and metastasis (8, 32). Prior studies have demonstrated that multiple C2H2-type zinc finger proteins participate in EMT regulation (4). For instance, ZEB1 and ZEB2 drive the EMT program by repressing E-cadherin transcription and are also involved in the maintenance of tumor stemness, therapeutic resistance and crosstalk with the tumor microenvironment, thereby acting as central regulators within the EMT network and contributing to chemoresistance in multiple cancer types (22, 23, 34). ZNF334 has been reported to inhibit EMT and stem-like phenotypes in triple-negative breast cancer through regulation of SFRP1 (24). Our transcriptomic analysis supported an association with EMT-related programs, whereas the Wnt/β-catenin GSEA result did not meet the stated significance criteria. ZNF114 knockdown impaired migratory and invasive abilities and shifted EMT markers toward an epithelial phenotype. The accompanying changes in Wnt-pathway proteins are consistent with an association between ZNF114 and Wnt/EMT-related processes, but do not establish its position within these pathways. However, whether ZNF114 directly regulates Wnt/EMT target genes or acts through interacting transcriptional complexes requires further mechanistic validation.

The tumor immune microenvironment plays a crucial role in gastric cancer progression and treatment response (27). EMT and Wnt activation have been repeatedly linked to reduced cytotoxic immune infiltration, impaired antigen presentation and immune exclusion (26, 33). In our analysis, ZNF114-high tumors showed a higher composite immune-cold index (FDR < 0.001), with individual immune-activation features (cytotoxic T/NK, T-cell-inflamed IFN, antigen presentation) showing downward trends that did not remain significant after FDR correction, alongside lower tumor mutational burden and a non-classical checkpoint pattern rather than a simple PDCD1/CD274/CTLA4-high phenotype. This distinction is important: the present data support an association between ZNF114 expression and the composite immune-cold index, but they do not demonstrate that ZNF114 directly regulates immune checkpoints or predicts immune checkpoint blockade response. The predicted drug-sensitivity analysis further provides therapy-related hypotheses, particularly around chemotherapy and pathway inhibitors, as EMT-associated signaling has been increasingly linked to chemoresistance in solid tumors (35), but these computational findings require validation in gastric cancer models.

Taken together, the functional experiments support a contribution of ZNF114 to malignant phenotypes in gastric cancer. Separately, high ZNF114 expression was associated with a higher composite immune-cold index in TCGA-STAD. These results support ZNF114 as a candidate prognostic biomarker and as a candidate molecule for future mechanistic and therapeutic studies, while emphasizing that direct targetability and treatment-predictive value remain to be established. Based on these data, we propose a hypothesis-generating working model in which ZNF114-high gastric cancer is linked to Wnt/EMT activation, a higher composite immune-cold index, altered checkpoint features, lower tumor mutational burden, and predicted drug-sensitivity differences (**Figure 10**). This model integrates the functional and computational results and provides a framework for future mechanistic and therapeutic studies.

**Figure 10:**
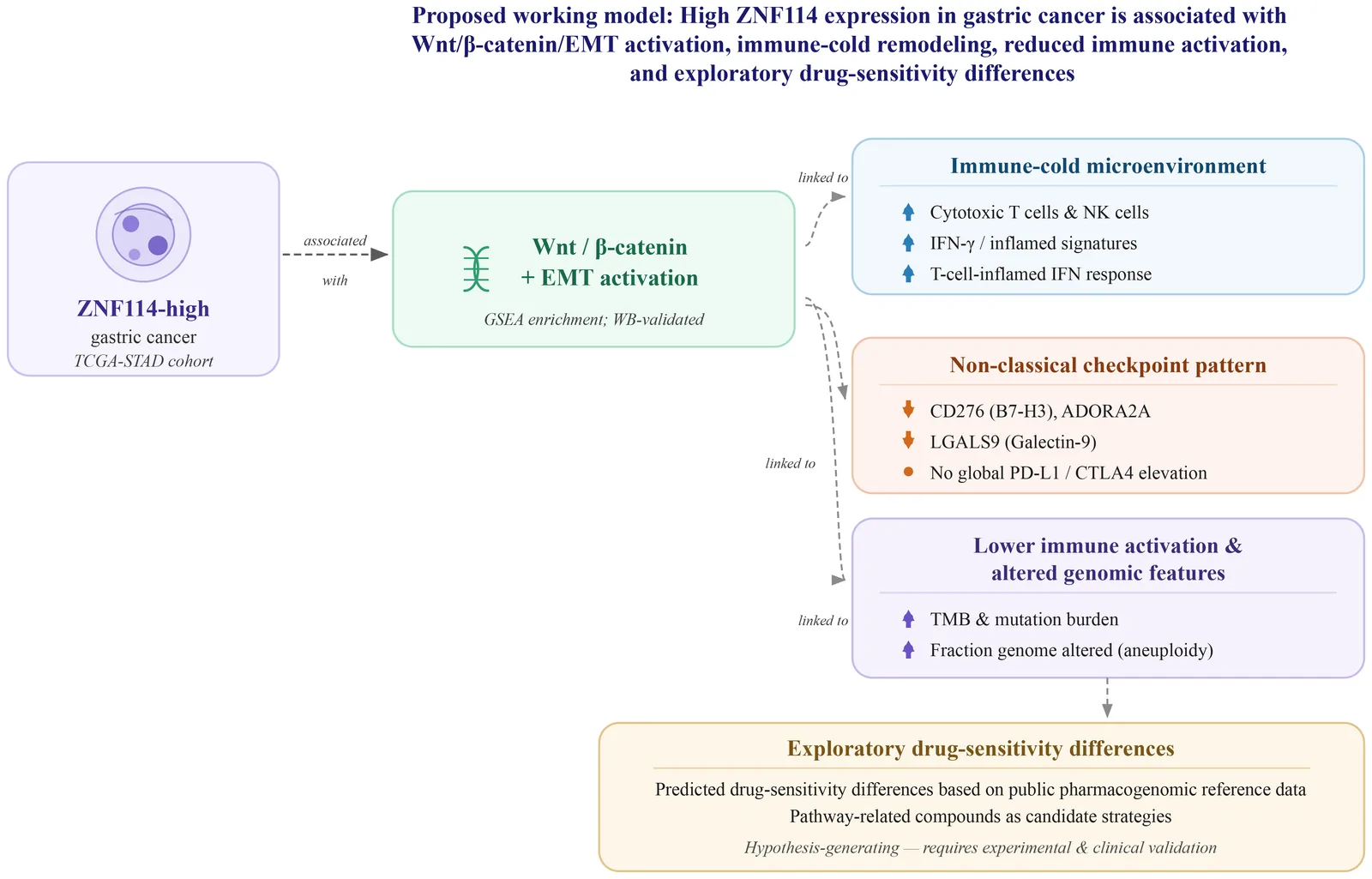
Working model of ZNF114-associated Wnt/EMT activation, immune-related transcriptomic features and therapy-related features in gastric cancer. The model integrates experimental and computational findings and should be interpreted as a framework for future mechanistic and therapeutic validation. The immune-related links represent observational associations and hypotheses for testing, not experimentally established causal pathways.

Several limitations should be considered when interpreting these findings. First, the immune-related and genomic findings are based on observational analysis of TCGA-STAD, whereas drug sensitivity was computationally predicted from TCGA-STAD expression profiles using a GDSC2-based model. No functional immune perturbation or direct validation of immune-cell abundance or spatial distribution was performed; these analysis do not establish that ZNF114 directly regulates immune checkpoints, causes immune exclusion or predicts immune-checkpoint blockade response. The shRNA xenograft experiments in immunodeficient nude mice support an effect on tumor growth but do not establish ZNF114-dependent regulation of antitumor immunity. Second, the observed immune-related associations overlap conceptually with established Wnt/β-catenin-associated immune-exclusion models (33) and reports linking other zinc-finger proteins to tumor–immune features (17); the contribution of the present work is the combined evaluation of ZNF114 expression and prognosis, its contribution to malignant cell phenotypes, and its exploratory immune-related and genomic associations in gastric cancer. A ZNF114-specific immune-exclusion mechanism, or a mechanistic distinction from established Wnt/EMT models, has not been demonstrated. Finally, the computational drug-sensitivity predictions are exploratory and require validation in gastric cancer models. In addition, the composite immune-cold index was not calibrated or externally validated against an established immune-hot/immune-cold classification and should therefore not be interpreted as a validated classifier; direct immune-cell profiling and functional immune assays remain necessary. The xenograft experiment also included five mice per group. This modest sample size provides supportive *in vivo* evidence but limits statistical power and generalizability; independent validation in larger cohorts will be needed. The clinical protein-level validation was limited to six patient-matched tissue pairs. Functional assays used AGS and HGC-27; the findings have not been established in MKN-45 or all gastric cancer cell models. Rescue experiments strengthen the specificity evidence, but a second independent siRNA was not carried through the functional assays. Direct regulation of Wnt/EMT targets has not been demonstrated.

Regarding therapeutic tractability, ZNF114 itself is a nuclear KRAB zinc-finger transcription factor and is currently a preclinical target hypothesis rather than a clinically validated drug target. Direct ZNF114-directed approaches such as siRNA, antisense oligonucleotides or targeted degradation face unresolved challenges in delivery, tumor penetration, pharmacokinetics and safety. The observed associations between ZNF114 expression and the CD276/B7-H3, ADORA2A and Wnt/β-catenin pathways suggest that downstream pathway or immune-modulator intervention may be a more feasible near-term translational strategy; however, these possibilities remain hypotheses to be tested in dedicated gastric-cancer preclinical models.

The present study focused on gastric cancer, and generalization of these findings to other tumor types—including microsatellite-stable colorectal cancer or immune-desert pancreatic cancer, in which Wnt/EMT-linked immune-exclusion features are also observed—awaits dedicated future studies in those specific disease contexts.

## 5 Conclusion

This study demonstrated that ZNF114 is markedly overexpressed in gastric cancer tissues and is associated with poor prognosis. Functionally, ZNF114 promotes gastric cancer cell proliferation, invasion and *in vivo* tumor growth, and is linked to Wnt/EMT activation. High ZNF114 expression is associated with a higher composite immune-cold index in TCGA-STAD with altered checkpoint features and predicted drug-sensitivity differences. Collectively, these findings suggest that ZNF114 is a candidate contributor to malignant progression with associated immune-related transcriptomic features in gastric cancer, warranting further mechanistic and therapeutic validation.

## Data Availability

The publicly available datasets analyzed in this study can be obtained from the following sources: The Cancer Genome Atlas (TCGA) stomach adenocarcinoma (STAD) dataset is available at https://portal.gdc.cancer.gov/; GEO datasets GSE79973, GSE65801, and GSE13861 are accessible at https://www.ncbi.nlm.nih.gov/geo/; Pan-cancer expression data were retrieved from TIMER3.0 (http://timer.cistrome.org/) and GEPIA (http://gepia.cancer-pku.cn/); Single-cell functional state analysis was performed using CancerSEA (http://biocc.hrbmu.edu.cn/CancerSEA/); The HPA-derived immunohistochemical images shown in **Figure 3G** were obtained from the Human Protein Atlas database (https://www.proteinatlas.org/). The in-house immunohistochemical images shown in **Figure 3L** were generated from the six paired clinical specimens described in Section 2.2 and are available from the corresponding author upon reasonable request. Data generated in this study: **Supplementary Tables S1**-**S4** provide the reported expression, index-composition and checkpoint statistics. The Bcl-2/Bax densitometry is provided in the source-data spreadsheet. Other original experimental data generated in this study, including Western blot images, CCK-8 assay results, colony formation data, flow cytometry data, Transwell assay results, immunohistochemical staining images, and animal experiment data, are available from the corresponding author (Wei Zhang, zzuzwys@163.com) upon reasonable request.

## Supplementary material

Supplementary Figure 1 Integrated screening workflow for ZNF114 candidate prioritization in gastric cancer. ZNF114 was prioritized through combined expression, prognostic and functional-state analysis and was selected for experimental validation.

Supplementary Figure 2 Specificity validation by si-2-resistant ZNF114 rescue. **(A)** Western blot analysis of ZNF114 protein expression in AGS and HGC-27 cells transfected with si-NC + empty vector, si-2 + empty vector, or si-2 + an si-2-resistant ZNF114 rescue construct. **(B)** Densitometric quantification of ZNF114 expression from panel A; values are normalized to si-NC. **(C, D)** Wound healing assays and quantification in AGS and HGC-27 cells under the same three conditions. **(E, F)** Colony formation assays and quantification. **(G–J)** Transwell migration and invasion assays and quantification. **(K)** Western blot analysis of E-cadherin, N-cadherin, Vimentin and GAPDH in AGS and HGC-27 cells under the same three conditions. In all panels, the rescue construct contained synonymous substitutions within the si-2-targeted sequence without altering the encoded ZNF114 protein sequence. Re-expression of ZNF114 attenuated the effects of si-2-mediated knockdown on wound closure, colony formation, Transwell migration/invasion and EMT-marker expression, providing complementary specificity evidence; however, because only a single siRNA sequence was used, the rescue data strengthen target-specificity evidence but do not establish a direct molecular mechanism. Original Western blot images are provided in WB Supplementary Information.
